# The effect of dehydroepiandrosterone supplementation on ovarian response is associated with androgen receptor in diminished ovarian reserve women

**DOI:** 10.1186/s13048-017-0326-3

**Published:** 2017-05-04

**Authors:** Qiaofei Hu, Liming Hong, Mingyue Nie, Qin Wang, Ying Fang, Yinmei Dai, Yanhong Zhai, Shuyu Wang, Chenghong Yin, Xiaokui Yang

**Affiliations:** 1 Department of Human Reproductive Medicine, Beijing Obstetrics and Gynecology Hospital, Capital Medical University, 251 Yaojiayuan Road, Chaoyang District, Beijing, 100026 China; 2 Department of Reproduction Regulation, Beijing Obstetrics and Gynecology Hospital, Capital Medical University, Beijing, 100006 China; 3 Department of Gynecology Minimally Invasive Center, Beijing Obstetrics and Gynecology Hospital, Capital Medical University, Beijing, 100006 China; 4 Department of Laboratory Medicine, Beijing Obstetrics and Gynecology Hospital, Capital Medical University, Beijing, 100026 China

**Keywords:** dehydroepiandrosterone/diminished ovarian reserve/androgen receptor/follicle stimulation hormone receptor/in vitro fertilization

## Abstract

**Background:**

Diminished ovarian reserve(DOR) is associated with female infertility and poor response to ovarian stimulation. Our objective was to assess the effect of dehydroepiandrosterone(DHEA) on DOR women and to explore whether the improvement of ovarian response after DHEA supplementation was dependent on the expression levels of androgen receptor(AR).

**Methods:**

A prospective cohort study was performed in the Department of Human Reproductive Medicine, Beijing Obstetrics and Gynecology Hospital during August 2014 to August 2016. 103 DOR women who completed the study were divided into the DHEA group (*n* = 53), which received DHEA supplementation (25 mg three times a day) for 8 weeks, and the control group (*n* = 50), which did not receive DHEA, before the IVF cycles. Serum hormone levels(FSH, LH, E_2_, T, DHEAs, AMH, INHB), antral follicle count(AFC) and the expression of AR and FSH receptor(FSHR) in granulosa cells(GCs) were measured, meanwhile ovarian response parameters and IVF outcomes were compared. The GCs from another 36 DOR women were cultured with different concentrations of DHEA in vitro. Then, we compared the expression of AR and FSHR in GCs according to the different numbers of oocytes retrieved both in DHEA and control group.

**Results:**

In the present study, DHEA supplementation resulted in significantly higher levels of serum T(*P* = 0.047), DHEAs(*P* = 0.019) and AR mRNA expression in GCs(*P* = 0.049). In vitro experiment, the protein and mRNA expression of AR and FSHR in the preovulatory GCs were significantly increased in response to DHEA supplementation(*P* <0.05). No significant differences were found in ovarian reserve, ovarian response, or IVF outcomes between the two groups. Subgroup analyses showed the levels of AR and FSHR mRNA in GCs were significantly increased in DHEA group with ≥5 oocytes retrieved(*P* <0.05).

**Conclusion:**

DHEA supplementation can increase the expression of AR in preovulatory GCs both in vivo and in vitro. The selective beneficial effects of DHEA supplementation on ovarian response in DOR women may depend on the increasing expression of AR and FSHR in GCs.

**Trial registration:**

The Chinese Clinical Trial Registry (ChiCTR-IPR-15006126). Retrospectively Registered 19 March 2015.

## Background

Ovarian reserve (OR), which refers to the quantity and quality of residual ovarian follicles and oocytes, reflects a woman’s reproductive potential and assisted reproductive technology (ART) outcomes [1, 2]. Women with diminished ovarian reserve (DOR) are associated with poor response to controlled ovarian stimulation (COS) resulting in fewer oocytes retrieval, poorer quality embryos and reduced pregnancy rates. DOR affects approximately 10% of infertile women [3]. Women with the following two or more items such as serum follicle stimulation hormone (FSH) level 25 IU/L > FSH > 10 IU/L, serum oestradiol (E_2_) level >80 pg/ml, serum anti-Mullerian hormone (AMH) level <0.5-1.1 ng/ml and antral follicle count (AFC) ≤5 on day 2-3 of a spontaneous cycle are considered to have DOR [4–6]. These women should be encouraged to conceive sooner due to their shorter fertility window [7]. Although various protocols have been used to improve pregnancy outcomes, infertility issues are still a challenge for DOR women.

It is believed that androgens could promote folliculogenesis and potentiate the effects of gonadotropin(Gn) [8, 9]. In recent years, dehydroepiandrosterone (DHEA), an endogenous steroid that primarily originates from the zona reticularis of the adrenal cortex and ovarian theca cells as well as circulating dehydroepiandrosterone sulfate (DHEAs) [10], has received increasing attention for its role in improving ovarian response and ART outcomes in DOR women [11–15]. Barad *et al* [11] reported that DHEA improved the ovarian response to COS in women with DOR and which was more evident in younger women[12]. A prospective self-controlled study indicated that the baseline ovarian reserve parameters such as AFC, FSH, E_2_, AMH and inhibin B (INHB) significantly improved after DHEA supplementation [13]. A randomized controlled trial (RCT) study reported that the accumulated score of embryos were significantly higher in DOR patients treated with DHEA compared to the control group [14]. However, the effect of DHEA supplementation on DOR women remains controversial. A meta-analysis including approximately 200 IVF cycles indicated that there was insufficient data to confirm the efficiency of DHEA supplementation in DOR or poor response women [16]. Similarly, Yeung et al [17] found that there was no statistically improvement in the ovarian reserve markers (AFC, AMH, or FSH), ovarian response to standard dose gonadotropin stimulation, or IVF outcomes in poor responders after DHEA pretreatment. So, there is a need for further study in order to provide more insights on the efficacy of DHEA.

The mechanism of actions following DHEA administration remains speculative. It has been reported that DHEA affects early follicle maturation by regulating androgen receptor (AR) transcription, increasing FSH receptor (FSHR) expression, modulating FSH activity in granulosa cells (GCs) and increasing the number of growing preantral and small antral follicles [18–22]. It was demonstrated that androgens could directly influence ovarian follicle health and development by AR-mediated actions using AR-knockout (ARKO) mouse model [23, 24]. Further study by Sen *et al* found that AR promoted follicle progression from pre-antral stage to the antral stage by a GCs-specific ARKO mouse model [25]. Despite the wide application of DHEA as an adjuvant therapy in women with DOR, its overall effectiveness and exact mechanism remain uncertain. We intended to examine whether DHEA supplementation affected the expression of AR and FSHR in GCs and what affected the different effects of DHEA pre-treatment in DOR women.

In the present study, we aimed to assess the effects of DHEA on ovarian reserve, ovarian response, and pregnancy outcomes of DOR women undergoing IVF treatment and to compare the expression of AR and FSHR in preovulatory granulosa cells both in vivo and in vitro and to investigate the relationship between AR, FSHR expression in granulosa cells and the numbers of oocytes retrieved in order to explore the potential benefits of DHEA supplementation.

## Methods

### Study population

In our study, participants gave their written informed consent after a detailed explanation regarding the meaning of ovarian deserve and information about the possible beneficial effects and potential side effects of DHEA. All procedures and sample collection methods were approved by the Human Ethics Committees of Beijing Obstetrics and Gynecology Hospital, Capital Medical University. The subfertile women undergoing IVF or intracytoplasmic sperm injection-embryo transfer (ICSI-ET) therapy were due to tubal and/or male factors or failure of intrauterine insemination (IUI) for 3 times. The inclusion criteria included [1] age <40 years, [2] duration of subfertility >1 year, and [3] DOR, diagnosis criteria including the following two or more items such as 25 IU/L > FSH >10 IU/L, E_2_ > 80 pg/ml, AMH <0.5-1.1 ng/ml and AFC ≤5 on day 2-3 of a spontaneous cycle. Patients were excluded if they had a diagnosis of polycystic ovary syndrome (PCOS), leiomyoma, endometriosis, metabolic disorders, or a history of ovarian or pelvic surgery, pelvic irradiation, hormonal treatment or DHEA supplementation.

### Study design

A prospective, cohort study was conducted from August 2014 to August 2016 in Department of Human Reproductive Medicine, Beijing Obstetrics and Gynecology Hospital. A total of 106 patients who understanded both the advantages and disadvantages of each method were divided into a treatment group and a control group according to whether they received DHEA (GNC LiveWell, Pittsburgh, PA, US) treatment before their first IVF cycles. In the DHEA treatment group (*n* = 54), patients were administered a 25-mg DHEA capsule three times daily for 8 weeks prior to their first IVF cycle. Another 52 patients comprised the control group. Of the 106 patients, 1 withdrew from the DHEA group, 1 was lost to follow-up and 1 went to another hospital in the control group. Accordingly, 103 patients completed the entire study (53 in the DHEA group and 50 in the control group). The preovulatory GCs from another 36 DOR patients were collected and cultured for in vitro experiment. This study was registered with the Chinese Clinical Trial Registry (ChiCTR-IPR-15006126).

### IVF regimen

DOR women received the Gonadotropin-releasing hormone (GnRH)-antagonist protocol. Exogenous gonadotropins (generally 150 to 225 IU/day) or Letrozole followed by 150 IU/d human menopausal gonadotropin (hMG) (Yantai Dongcheng Beifang Pharmaceutical Co., Shandong Province, China) were administered until the follicles reached maturity. GnRH-antagonists (Cetrotide 0.25 mg, Qd, STAMedica, Amsterdam, Netherlands) were initiated when the largest follicle reached a diameter of 13-14 mm. Human chorionic gonadotropin (hCG) (250 μg, Merck Serono Inc., Geneva, Switzerland) was administered when the leading follicle reached a diameter of 18 mm or at least two follicles reached 17 mm in diameter. Oocyte retrieval was performed 34-36 h after hCG administration. A maximum of two embryos were transferred 2-3 days after retrieval. Luteal support was started on the day following oocyte retrieval and continued for an additional 9-10 weeks. The achievement of a biochemical pregnancy was assessed by the serum hCG assay 14-16 days after the embryo transfer. A clinical pregnancy was defined as visualization of a gestational sac under ultrasonography 30-35 days after the embryo transfer.

### Assay of serum hormonal levels and AFC

Serum was collected on Day 2-3 in the menstrual period before and after DHEA treatment prior to ovulation induction. All samples were stored at -80 °C until they were assayed. Serum samples were assayed for AMH, INHB, DHEAs, FSH, E_2_, T, and luteinizing hormone (LH). Serum basal FSH, LH, E_2_ and T were measured using the Beckman Coulter Access II Immunoassay system, and basal DHEAs was measured using the DHEAs Enzyme-linked Immunosorbent Assay Kit(ELISA) kit (Huaxing Bio Biotechnology, Beijing, China). Serum AMH and INHB levels were measured using an AMH and INHB ELISA Kits (Ansh Labs, Texas, Houston, UK). All experiments were performed according to the manufacturer’s instruction. The intra-assay coefficients of variation (CVs) were less than 10% for AMH and INHB, less than 9% for DHEAs. The inter-assay CVs were less than 15% for AMH, INHB and DHEAs. The detection range was 0.24-11.78 ng/ml for AMH, 19.67-147.62 pg/ml for INHB and 8-400 μg/dl for DHEAs. The AFC in both ovaries were measured by trans-vaginal ultrasonography scans on day 2-3 of menstrual cycle in all patients.

### Isolation and collection of granulosa cells

Human granulosa cells were collected from the follicular fluid of dominant follicles (follicle diameter ≥ 14 mm) undergoing oocyte retrieval and purified by density centrifugation from follicle aspirations, as described by Nie et al. and Shi et al [26, 27]. Each woman’s cells were processed separately and were not mixed. The aspirates were centrifuged at 400 *g* for 10 min at room temperature and follicular fluid was removed. Granulosa cells were separated from red blood cells and others by centrifugation through 50% Percoll (Sigma, Saint Louis, MO, USA) for 15 min at 1000 *g*. The GCs in the intermediary layer were harvested. Following centrifugation and washing with phosphate-buffered saline (PBS), the cells were resuspended in RPMI-1640 containing 10% foetal bovine serum (FBS) and 1% antibiotic-antimycotic (penicillin G, streptomycin, and amphotericin B; Sigma), then centrifuged at 400 *g* for 2 min at room temperature. The supernatant was discharged and the GCs were placed at -80 °C for RNA extraction.

### In vitro culture of primary granulosa cells by DHEA

Preovulatory GCs from another 36 DOR patients without DHEA supplementation were purified, pooled and seeded in 6-well or 12-well plates to ensure that they attained 40–50% confluence by the next day. Then, GCs were treated with a DMSO control or different concentrations of DHEA (10, 20, 30, 40, or 50 ng/ml) (Sigma) for 24 or 48 h in a humidified atmosphere of 5% CO_2_ at 37 °C. The cultured GCs were then removed from the dishes after treatment with 0.25% trypsin at 37 °C and collected by centrifugation.

### RNA extraction and quantitative RT-PCR

Total RNA was extracted from human ovarian GCs using the TRIzol reagent (Invitrogen, Shanghai, China) according to the manufacturer’s instructions. cDNA was synthesized using 2 μg of RNA as previously reported [28]. Quantitative RT-PCR was performed using a SYBR Green kit (Takara Bio Inc., Shiga, Japan), according to the manufacturer’s instructions. Each 20-μl reaction mixture included 2 μl of cDNA, 10 μl of SYBR Green Mix, 1.6 μl of the specific primer, and 6.4 μl of RNase-free water. The following specific primers (Invitrogen, Shanghai, China) were used for the AR [29] and FSHR [30]: AR, 5’- TTG TCC ATC TTG TCG TCT TCG G-3’ (forward) and 5’-TGTCCAGCACACACTACACC-3’(reverse); FSHR, 5’-GG TGC ATT TTC AGG ATT TGG G-3’ (forward) and 5’-TTG GGA AGG TTG GAG AAC AC-3’ (reverse). GAPDH [30] was an endogenous control (5’-TGT TGC CAT CAA TGA CCC CTT-3’ [forward] and 5’-CTC CAC GAC GTA CTC AGC G-3’ [reverse]). qRT-PCR was performed on an Applied Biosystems 7500 Fast Real-Time PCR System (Applied Biosystems, Carlsbad, CA). The PCR reactions were conducted at 95 °C for 30 s, followed by 40 cycles at 95 °C for 5 s, 60 °C for 20 s, and 72 °C for 15 s. The specificity of the PCR products was confirmed by analysis of the dissociation curve. The relative concentration of each mRNA was calculated according to the formula 2–ΔΔCt. All of the reactions were performed in triplicate, and all experiments were repeated three times.

### Western blot analysis

Western blot analysis was performed as described by Yang et al [28]. Briefly, the total proteins were extracted from human ovarian GCs using whole-cell lysis buffer (4 mM EGTA, 3 mM EDTA, pH 8.0, 0.5% NP40, 12.5 mM HEPES, 1 mM DTT, 0.5 mM Na_3_VO_4_, 125 mM NaF, 2.5 mg/ml aprotinin, 25 mg/ml trypsin inhibitor, and 25 mM PMSF). The protein concentrations were determined using a BCA Protein Assay Kit (Pierce, Rockford, IL, USA). A total of 20 μg of the total protein was separated by sodium dodecyl sulfate-polyacrylamide gel electrophoresis (SDS-PAGE), followed by electrotransfer onto nitrocellulose membranes. The membranes were blocked with 5% skim milk for 1 h and incubated overnight at 4 °C with specific primary antibodies (anti-AR: 1:1000, No. 5153, Cell Signaling Technology Inc.; anti-FSHR: 1:1000, No. H-190, Santa Cruz Biotechnology Inc.; anti-GAPDH: 1:1000, No. ab22556, Abcam, Hong Kong, China). After washing the membranes three times with 0.1% Tween in Tris-buffered saline (TBST), the membranes were incubated with horseradish peroxidase (HRP)-conjugated secondary antibodies (Cell Signaling Technology Inc. Danvers, MA, US) at room temperature for 1 h. The reactive bands were visualized using an enhanced chemiluminescence kit (Thermo Fisher Inc. Shanghai. China). The final results were analysed using Scion Image software (http://scion-image.software.informer.com/).

### Statistical analysis

All data were analysed using SPSS 21.0 software (IBM Corp., Armonk, NY, USA). The results are expressed as the means ± standard deviations (SDs), and normally distributed data were compared by means of independent samples t-tests. Non-normally distributed data were compared using the Mann-Whitney *U* test. The chi-square test was used to compare fertilization, embryo quality, embryo implantation, and clinical pregnancy rates between the two groups. Bonferroni adjustment was for multiple comparisons. The Spearman correlation coefficient was used to assess the relationship between T, DHEAs and other variables. A *P* value <0.05 was considered to be of statistical significance.

## Results

### Baseline characteristics of DOR patients in the DHEA and control groups

Among the 106 women eligible based on the inclusion criteria, 103 completed the study. Of these patients, 53 and 50 were allocated to the DHEA group and control group, respectively. Basic demographic characteristics, such as age, BMI, age of menarche, duration of infertility, abortion number, infertility aetiology, basic hormone levels (FSH, LH, E_2_, T, P, AMH, INHB, and DHEAs) and AFC, were not significantly different (*P* >0.05) between the DHEA and control groups (Table 1).

**Table 1:** Baseline characteristics of DOR women in DHEA group and control group(Mean ± SD)

Variables	DHEA group (*n* = 53)	Control group (*n* = 50)	P
Age(year)	33.28 ± 3.13	34.16 ± 3.27	0.194^b^
BMI(kg/m^2^)	22.32 ± 2.44	23.24 ± 4.41	0.444^b^
the age of menarche(year)	13.19 ± 1.37	12.98 ± 1.19	0.343^b^
Duration of Infertility(year)	3.81 ± 2.61	3.86 ± 2.56	0.838^b^
Type of subfertility			
—Primary	22(41.51%)	24(48.0%)	0.508^c^
—Secondary	31(58.49%)	26(52.0%)	
Causes of subfertility			
—Tuboperitoneal	22(41.51%)	17(34.0%)	0.752^3*^
—Male	14(26.42%)	14(28.0%)	
—Mixed	11(20.75%)	10(20.0%)	
—IUI failure	6(11.32%)	9(18.0%)	
Number of abortion	0.45 ± 0.77	0.40 ± 0.88	0.510^b^
FSH(IU/L)	10.57 ± 1.91	10.45 ± 2.27	0.190^b^
LH(IU/L)	3.82 ± 1.48	3.80 ± 1.68	0.566^b^
FSH/LH	3.19 ± 1.34	3.15 ± 1.25	0.840^b^
E_2_(pg/ml)	46.83 ± 23.86	47.46 ± 23.66	0.947^b^
T(ng/dl)	23.36 ± 15.45	25.11 ± 13.04	0.278^b^
Inhibin B(pg/ml)	41.18 ± 12.74	39.51 ± 10.48	0.471^a^
AMH(ng/ml)	0.87 ± 0.20	0.88 ± 0.20	0.840^b^
DHEAs(μg/dl)	13.60 ± 4.09	14.30 ± 3.80	0.243^b^
AFC	4.25 ± 1.45	4.40 ± 1.21	0.359^b^

^a^independent samples t-tests, ^b^Mann-Whitney *U* test, ^c^ *X* ^2^ test, *Bonferroni adjustment *P*’ >0.008

### Effects of DHEA supplementation on ovarian reserve, ovarian response and IVF outcomes

Serum hormonal levels and AFC were determined after 8 weeks of DHEA supplementation. As shown in Table 2, serum T and DHEAs levels were significantly increased in the DHEA treatment group compared with those in the control group (*P* <0.05). The serum FSH level (9.51 ± 1.78 IU/L) decreased, while AFC (5.34 ± 1.68) along with the levels of AMH (0.96 ± 0.16 ng/ml) and INHB (49.01 ± 15.56 pg/ml) increased in the DHEA group; however, there were no significant differences between the DHEA and control groups (*P >0.05)* (Table 2).

**Table 2:** Comparison of ovarian reserve and ovarian response parameters and IVF outcomes after DHEA supplementation(Mean ± SD)

Items	DHEA (*n* = 53)	group control group (*n* = 50)	P
Treatment variables			
T(ng/dl)	34.02 ± 19.02	25.58 ± 13.59	0.047^**b**^
DHEAs(d2-3) (μg/dl)	16.52 ± 4.30	14.71 ± 4.26	0.019^**b**^
Ovarian reserve variables			
FSH(IU/L)	9.51 ± 1.78	10.16 ± 2.48	0.083^**b**^
Inhibin B(d2-3)(pg/ml)	49.01 ± 15.56	45.53 ± 14.91	0.074^**b**^
AMH(ng/ml)	0.96 ± 0.16	0.89 ± 0.20	0.058^**a**^
AFC	5.34 ± 1.68	4.66 ± 1.44	0.085^**b**^
Ovarian response variables			
Total doses of Gn (IU)	2292.45 ± 842.27	2555.00 ± 963.94	0.151^b^
Stimulation duration(days)	9.79 ± 1.71	10.18 ± 1.90	0.410^b^
E_2_ on hCG day(pg/ml)	1903.28 ± 1178.43	1578.93 ± 956.63	0.339^b^
Endometrial thickness on hCG day(mm)	9.73 ± 1.66	9.29 ± 2.11	0.245^a^
N of retrieved oocytes	4.51 ± 2.40	3.70 ± 2.00	0.098^b^
N of fertilization oocytes	3.75 ± 2.32	3.02 ± 1.95	0.084^b^
N of embryos transferred	1.78 ± 0.49	1.88 ± 0.49	0.425^b^
High quality embryos rate(%)	60.26(94/156)	58.27(74/127)	0.735^c^
Pregnancy outcomes			
Implantation rate of embryo(%)	23.08(15/65)	21.67(13/60)	0.850^c^
Clinical pregnancy rate(%)	33.33(12/36)	28.13(9/32)	0.643^c^
Early abortion rate(%)	0(0/12)	22.22(2/9)	0.171^c^
Ectopic pregnancy rate(%)	0(0/12)	22.22(2/9)	0.171^c^

^a^independent samples t-tests, ^b^Mann-Whitney *U* test, ^c^ *X* ^2^ test

Characteristics of IVF cycles regarding the COS and IVF outcomes were observed. With DHEA supplementation, the levels of E_2_ (1903.28 ± 1178.43 pg/ml vs. 1578.93 ± 956.63 pg/ml) and endometrial thickness on the hCG day (9.73 ± 1.66 mm vs. 9.29 ± 2.11 mm) were increased, while the total Gn dose (2292.45 ± 842.27 IU vs. 2555.00 ± 963.94 IU) and Gn stimulation duration (9.79 ± 1.71 d vs. 10.18 ± 1.90 d) were decreased. There were increased numbers of retrieved oocytes (4.51 ± 2.40 vs. 3.70 ± 2.00), higher rates of embryo implantation (23.08%) and clinical pregnancy (33.33%) in the DHEA group, although there were no significant differences between the DHEA and control groups (Table 2). With DHEA supplementation, the rates of early abortion and ectopic pregnancy were decreased, but no significance (Table 2).

### Comparison of the expression of AR mRNA and FSHR mRNA in the GCs of DOR women

**Fig. 1:**
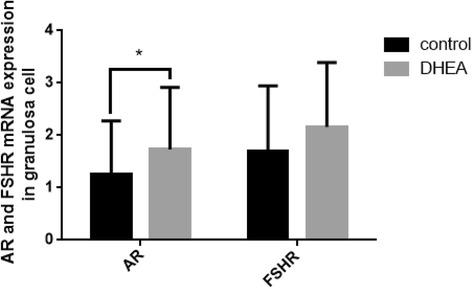
Quantitative analysis of AR and FSHR mRNA levels in preovulatory GCs collected at oocyte retrieval. The numbers of collected GCs were 30 in DHEA and 29 in control group. Each bar represents the mean ± SD. Concomitant detection of GADPH mRNA in the real-time RT-PCR reaction served as a reference for relative quantification. Data were the mean values ± SD and all experiments were repeated three times (* *P < 0.05*)

We next detected the expression levels of AR mRNA and FSHR mRNA in preovulatory GCs collected from corresponding follicular fluid of DOR patients so as to find whether their expression was influenced by DHEA pre-treatment during COS. The mRNA levels of the AR and FSHR were quantified by real-time PCR and the relative levels were compared between the DHEA group (*n* = 30) and the control group (*n* = 29). The results were summarized in Fig. 1. The expression of AR mRNA in GCs was significantly higher in DHEA group (*p* = 0.049). FSHR mRNA expression increased in DHEA group but there was no significant difference between the two groups (*p* = 0.064).

### DHEA upregulated the AR and FSHR mRNA and protein expression in the GCs in vitro

**Fig. 2:**
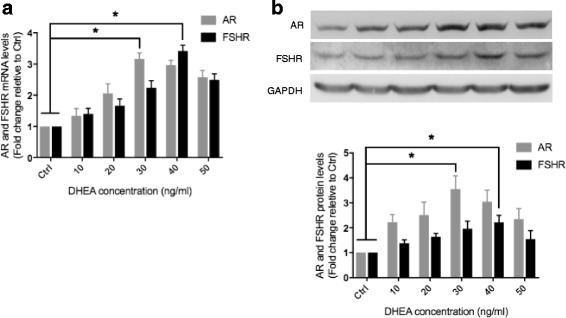
DHEA up-regulates AR and FSHR expression in preovulatory GCs in vitro. Granulosa cells collected from another 36 DOR women without DHEA supplementation before IVF/ISCI were treated with DMSO (Ctrl) and different concentrations (10, 20, 30, 40, 50 ng/ml) of DHEA. mRNA (Fig. 2A.) and protein (Fig. 2b.) of AR and FSHR were collected at 24 h and 48 h after DHEA treatment and examined using RT-qPCR and Western blot respectively. Data were the mean values ± SD and all experiments were repeated three times (* *P < 0.05*)

To further demonstrate the effects on AR and FSHR levels in GCs after DHEA pretreatment, we assessed the expression of AR and FSHR on mRNA and protein levels in cultured preovulatory GCs. The GCs collected from another 36 DOR patients without DHEA supplementation before IVF were pooled together and seeded on 6 or 12-well plates and then treated with DHEA at different concentrations (10, 20, 30, 40, 50 ng/ml) for 24-48 h, DMSO as control. We observed that the AR mRNA and protein expression levels increased with increasing DHEA concentration. The expression of AR reached the highest level at 30 ng/ml DHEA, and subsequently decreased (Fig. 2a, b). Similarly, the expression of FSHR reached the highest levels with 40 ng/ml DHEA (Fig. 2a, b).

### Correlation analysis between the serum T, DHEAs levels and the characteristics, the ovarian response and the expression of AR and FSHR mRNA in GCs

Studies have shown that hypoandrogenism is associated with diminished functional ovarian reserve (DFOR) and that DHEA supplementation augments ovarian stimulation in poor responders [9, 31]. As we determined previously, T and DHEAs levels were increased after DHEA supplementation. To study the effects of androgens on ovarian reserve and ovarian response in DOR patients undergoing IVF treatment, the correlation between the serum T and DHEAs levels and the ovarian response, expression of AR and FSHR mRNA during ovarian stimulation was analysed in our study. The serum T levels were positively correlated with the number of retrieved oocytes (*r* = 0.457), high-quality embryos (*r* = 0.462), AFC(*r* = 0.310), E_2_ on HCG day (*r* = 0.215), AR mRNA expression (*r* = 0.769) and FSHR expression (*r* = 0.616), and were inversely related to the age (*r* = -0.253). The serum DHEAs levels were positively correlated with the number of retrieved oocytes (*r* = 0.579), AR mRNA expression (*r* = 0.362) and FSHR expression (*r* = 0.320), and were negative correlated with bFSH (*r* = -0.245) (Table 3).

**Table 3:** Correlation analysis between serum T, DHEAs and ovarian response, AR and FSHR mRNA levels

Variables	testosterone		DHEAs	
	r	P	r	P
Age	-0.253	0.010	-0.002	0.982
bFSH	-0.115	0.247	-0.245	0.013
bAFC	0.310	0.001	0.180	0.068
E_2_(HCG)	0.215	0.029	0.133	0.181
Total doses of Gn	0.069	0.489	-0.032	0.746
Stimulation duration(d)	0.096	0.337	0.076	0.446
N of retrieved oocytes	0.457	< 0.0001	0.579	< 0.0001
N of High quality embryos	0.462	< 0.0001	0.183	0.064
AR mRNA	0.769	< 0.0001	0.362	0.005
FSHR mRNA	0.616	< 0.0001	0.320	0.013

Spearman correlation

### AR mRNA and FSHR mRNA expression in the GCs was associated with ovarian response

**Fig. 3:**
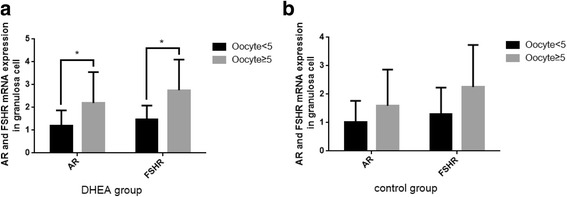
The DHEA group (n = 30) and the control group (n = 29) were divided into subgroups based on the number of oocytes retrieved (≥5 or <5) respectively. The expression of AR and FSHR mRNA in DHEA group with more oocytes retrieved (n = 16) were significantly increasing than those with less oocytes retrieved (n = 14) (*P < 0.05). The expression of AR and FSHR didn’t reach statistically significant difference in control group (P > 0.05), and the number of collected GCs was 12 cases in oocytes retrieved ≥5 group and 17 cases in oocytes retrieved <5 group. AR and FSHR mRNA were examined using RT-qPCR. Data were the mean values ± SD and all experiments were repeated three times

From the present study, we found serum T, DHEAs levels and AR mRNA expression were significantly increased after DHEA supplementation, and serum T and DHEAs levels positively correlated with the number of oocytes retrieved and expression of AR mRNA. To study the correlation between the effects of DHEA on the ovarian response and expression of AR and FSHR mRNA, we further assessed the AR and FSHR mRNA expression in DHEA and control groups. The DHEA group was divided into two subgroups (A and B) based on the number of oocytes retrieved. The number of retrieved oocytes was ≥5 in group A (*n* = 16) and <5 in group B (*n* = 14). As shown in Fig. 3a, the expression of AR and FSHR mRNA in GCs in group A were significantly higher than those in group B (*P <0.05*). The control group was also divided into two subgroups (C and D) based on the number of oocytes retrieved. The number of retrieved oocytes was ≥5 in group C (*n* = 12) and <5 in group D (*n* = 17). The expression of AR and FSHR mRNA increased, but they didn’t reach significant difference (*p* = 0.268 and p = 0.057) (Fig. 3b).

## Discussion

Diminished ovarian reserve is associated with a poor ovarian response (POR) to gonadotropin stimulation and IVF outcomes. Whether pretreatment with DHEA improves the ovarian response in DOR patients remains debated. In the present study, no statistically significant improvement in ovarian reserve markers, ovarian response, or IVF outcomes was found. However, subgroup analysis showed that the expression of AR and FSHR mRNA in GCs were significantly increased in women with ≥5 oocytes retrieved after DHEA pretreatment, suggesting that DHEA may improve the ovarian response in DOR women through promoting the expression of AR and FSHR in GCs.

Although many studies have focused on the effects of androgens and AR on follicular development, the conclusions are controversial. A recent meta-analysis indicated the clinical pregnancy rate was statistically increased in DOR patients who were pre-treated with DHEA, there was no significant improvement on the number of oocytes retrieved and the cancellation rate, and there was a non-significant difference in the clinical pregnancy rate when data were restricted to RCTs [32]. DHEA is diverted from DHEAs in GCs after removing the sulfate by steroid sulfatase [33]. The concentrations of both DHEA and DHEAs decrease progressively with age [34]. DFOR which represents an androgen deficiency condition is associated with significant hypoandrogenism at all ages and it gains a benefit from DHEA supplementation [31]. Androgens promote the recruitment and initiation of primordial follicles, induce the development of primary, preantral, and antral follicles through the upregulation of AR, FSHR, and insulin-like growth factor-1 (IGF-1) in GCs, induce paracrine regulation, and reduce follicle atresia [8, 20, 35, 36]. In our study, oral supplementation with DHEA for 8 weeks significantly increased serum T and DHEAs levels (Table 2), and produced a slightly improvement on AFC, FSH and AMH levels, suggesting that DHEA supplementation may lead to higher T levels, readily providing a pool of ovarian steroidogenic prohormones and promoting follicular growth. Meanwhile, the Gn doses and stimulation days decreased, while the E_2_ levels on hCG day, the numbers of oocyte retrieval and fertilization slightly increased after DHEA pretreatment. It appeared that DHEA had a positive effect on ovarian response in DOR women and we need a large sample research to verify the efficiency. Gleicher N et al [37] reported a significant reduction in the number of aneuploidy embryos after DHEA supplementation. But, currently our results didn’t support the conclusion that DHEA supplementation improved embryo quality (Table 2).

The action of androgen is mediated by AR, as a member of nuclear receptor superfamily encoded by an X chromosomal gene, interacts directly with target genes to regulate follicle development and function [38, 39]. Meanwhile, the beneficial effects of androgens on follicle maturation are mediated through the AR on granulosa cells in small growing follicles [25]. ARs are widely expressed throughout the whole development of folliculogenesis in the oocytes, GCs, theca cells, and stromal cells, and the expression of ARs is abundant in the preantral and antral stages of follicular development then declines as follicles mature to the preovulatory stage [19, 40]. A study [21] in primates proved that androgens (testosterone) increased AR expression in the theca and GCs of preantral follicles. But whether DHEA can improve the AR and FSHR expression in the GCs from preovulatory follicles of DOR women is still unknown. Using luteinized GCs in preovulatory follicles, we found significantly increasing expression of AR mRNA in DHEA group which indicated that DHEA supplementation may upregulate the AR expression in preovulatory GCs of DOR women (Fig. 1). Studies uniformly suggest that androgens can increase the levels of FSHR in different species [20, 35, 41, 42], however, in our study the expression of FSHR mRNA in GCs was higher in DHEA group compared to the control group, there was no significantly difference between the two groups (Fig. 1).

To further investigate the regulation action of DHEA on AR and FSHR, we cultured preovulatory GCs from another 36 DOR women with different concentrations of DHEA in vitro. AR and FSHR mRNA and protein expression was increased in a concentration-dependent manner (Fig. 2). The isolated human granulosa cells were found to show a significant upregulation of AR and FSHR in both the mRNA and protein levels with DHEA treatment. It seemed that DHEA got metabolized to testosterone, and that the positive effects of DHEA supplementation are mediated through the AR. These finding suggest that DHEA may improve AR and FSHR expression in preovulatory GCs of DOR in vitro.

In the present study, we found positive correlations between the serum T, DHEAs and oocytes retrieved, the expression of AR and FSHR mRNA (Table 3), indicating that women with higher levels of T and DHEAs may have a greater number of retrieved oocytes and higher expression of AR and FSHR mRNA. Whether the effect of DHEA on ovarian response was achieved through the increasing expression of AR in GCs remained unclear. To confirm the relationship between the number of oocytes retrieved and the expression of AR and FSHR in GCs, subgroup analysis were performed by dividing the DHEA group and control group according to the number of oocytes retrieved (≥5 or <5; Fig. 3). Expression of AR and FSHR mRNA in GCs was significantly increased in DHEA group with ≥5 oocytes retrieved, while these expressions didn’t reach statistical difference in control group (Fig. 3). It indicates that the expression of AR and FSHR was associated with increased ovarian response after DHEA supplementation. The DOR women who benefited from DHEA supplementation exhibited an enhanced expression of AR and FSHR. Therefore, the upregulation of AR and FSHR may be a possible mechanism to improve follicle growth and responsiveness in some DOR women with DHEA treatment.

## Conclusion

In conclusion, DOR women who received DHEA supplementation display increased expression of AR mRNA in GCs, suggesting that DHEA pretreatment upregulate the AR expression. The selective beneficial effect of DHEA on ovarian response in DOR women may be associated with the upregulation of AR and FSHR.
